# Evolution of resistance to single and combined floral phytochemicals by a bumble bee parasite

**DOI:** 10.1111/jeb.13002

**Published:** 2016-11-23

**Authors:** E. C. Palmer‐Young, B. M. Sadd, L. S. Adler

**Affiliations:** ^1^ Department of BiologyUniversity of Massachusetts at AmherstAmherstMAUSA; ^2^ School of Biological SciencesIllinois State UniversityNormalILUSA

**Keywords:** *Bombus*, cell culture, *Crithidia bombi*, dose–response curves, drug resistance, EC50, eugenol, experimental evolution, Markov chain Monte Carlo, thymol

## Abstract

Repeated exposure to inhibitory compounds can drive the evolution of resistance, which weakens chemical defence against antagonists. Floral phytochemicals in nectar and pollen have antimicrobial properties that can ameliorate infection in pollinators, but evolved resistance among parasites could diminish the medicinal efficacy of phytochemicals. However, multicompound blends, which occur in nectar and pollen, present simultaneous chemical challenges that may slow resistance evolution. We assessed evolution of resistance by the common bumble bee gut parasite *Crithidia bombi* to two floral phytochemicals, singly and combined, over 6 weeks (~100 generations) of chronic exposure. Resistance of *C. bombi* increased under single and combined phytochemical exposure, without any associated costs of reduced growth under phytochemical‐free conditions. After 6 weeks’ exposure, phytochemical concentrations that initially inhibited growth by > 50%, and exceeded concentrations in floral nectar, had minimal effects on evolved parasite lines. Unexpectedly, the phytochemical combination did not impede resistance evolution compared to single compounds. These results demonstrate that repeated phytochemical exposure, which could occur in homogeneous floral landscapes or with therapeutic phytochemical treatment of managed hives, can cause rapid evolution of resistance in pollinator parasites. We discuss possible explanations for submaximal phytochemical resistance in natural populations. Evolved resistance could diminish the antiparasitic value of phytochemical ingestion, weakening an important natural defence against infection.

## Introduction

Effective medicinal compounds, whether natural or synthetic, are vulnerable to the evolution of resistance by the parasites that they target. The clinical significance of resistance to antibiotics is considered a major threat to human health (Bonhoeffer *et al*., 1997). In agriculture, resistance to pesticides has created an ongoing need for new means of genetic and chemical control (Barrett & Antonovics, 1988; Bates *et al*., 2005). Similarly, in natural systems, the evolution of phytochemical resistance by specialist antagonists necessitates the biosynthetic invention of new plant defences (Berenbaum & Feeny, 1981) by diminishing the effectiveness of originally toxic compounds. For example, specialist herbivores such as *Manduca sexta*, which specialize on *Nicotiana* plants, have higher resistance to nicotine than do related Lepidopterans (Wink & Theile, 2002); and monarch butterflies are 300‐fold more resistant than other Lepidopterans to the cardiac glycosides of their host milkweed plants (Vaughan & Jungreis, 1977). Repeated or chronic exposure to inhibitory phytochemicals exerts strong positive selection for resistance (Elfawal *et al*., 2015), which can attenuate the effectiveness of the chemical and create the need for additional compounds or higher doses to achieve the same effect (Read *et al*., 2011).

Plants produce an astounding diversity of phytochemicals that can counteract infection in the plants themselves and also in phytophagous animals that consume phytochemicals (Hartmann, 2007; de Roode *et al*., 2013). Flowers contain distinct phytochemicals and blends that structure surface microbial communities (Junker *et al*., 2011) and can protect flowers from infection (Huang *et al*., 2012). Ingestion of antimicrobial phytochemicals may also ameliorate disease in phytophagous animals. Many animals prefer and seek out particular plants and phytochemicals when infected; ingestion of phytochemical‐rich plants and their constituent compounds may reduce levels of infection (de Roode *et al*., 2013). Among insects, generalist arctiid caterpillars sought out alkaloid‐containing host plants when parasitized; consuming these hosts increased the chances of surviving parasitism (Karban & English‐Loeb, 1997; Singer *et al*., 2009). Cardenolide‐rich latex from *Asclepias* improved survival and reduced spore counts of monarch butterfly larvae inoculated with protozoa (Gowler *et al*., 2015). Like foliage consumed by herbivores, nectar, pollen and other plant products used by pollinators are rich in antimicrobial phytochemicals (Dobson & Bergstrom, 2000; Heil, 2011). In honey bees, gathering of resins reduced chalkbrood infection (Simone‐Finstrom & Spivak, 2012); consumption of plant‐derived honeys (Gherman *et al*., 2014) and the floral phytochemical thymol reduced levels of the microsporidian parasite *Nosema ceranae* (Costa *et al*., 2010). Certain nectar phytochemicals also ameliorated *Crithidia bombi* infection in bumble bees (Manson *et al*., 2010; Baracchi *et al*., 2015; Richardson *et al*., 2015). The strong effects of phytochemicals on plant and animal parasites may impose selective pressures that could drive the evolution of phytochemical resistance in frequently exposed parasite populations.

The evolution of parasite resistance to natural or artificial compounds could exacerbate the negative impacts of parasites and pathogens on pollinators. Resistance of *Varroa* mites, which parasitize honey bees, has decreased the effectiveness of conventional miticides in apiculture (Lodesani *et al*., 1995; Rosenkranz *et al*., 2010). Phytochemical miticides, such as thymol (Giacomelli *et al*., 2015) and eugenol (Maggi *et al*., 2010), have emerged as natural alternatives to acaricides (Rosenkranz *et al*., 2010). However, the recommended treatment regime, consisting of repeated and prolonged administration of phytochemicals (Imdorf *et al*., 1996), results in incomplete eradication of the mites (Gregorc & Planinc, 2005), thereby providing conditions under which phytochemical resistance could evolve. In addition, the persistence of prophylactic chemicals at weakly inhibitory concentrations in hive materials (Nozal *et al*., 2002; Floris *et al*., 2004) may continue to select for resistant genotypes, even after treatment is complete. Even in the absence of deliberate prophylactic treatment with phytochemicals, chronic exposure to the environmental phytochemicals could create sufficient selective pressure to favour phytochemically resistant parasites. This problem is especially relevant in agricultural landscapes with intentionally low floral diversity, where one or two species may cover the majority of land within a 2‐km radius (Long & Krupke, 2016). Low floral diversity is likely to result in a correspondingly low phytochemical diversity in available nectar and pollen. Monotonous exposure of parasites to these chemicals could give rise to chemically resistant parasite populations, thereby reducing the medicinal value of the few compounds available in monocultures. For example, the bumble bee gut parasite, *C. bombi*, is over 100‐fold more resistant to several phytochemicals than are phylogenetically related trypanosomes vectored by blood‐feeding insects (Palmer‐Young *et al*., accepted). The high resistance of *C. bombi,* which has more direct exposure to floral phytochemicals than do related trypanosomes, suggests that phytochemical resistance can be increased by exposure to nectar and pollen phytochemicals over evolutionary time.

Whereas monotonous exposure to single chemicals creates strong selection for resistance, chemical combinations are thought to retard the evolution of resistance (Hastings, 2011), and associated costs may curtail the spread of resistance in populations. Pollinator parasites are likely to be frequently exposed to phytochemical combinations when their hosts consume nectar and pollen from multiple plant species or phytochemical blends produced by a single species. For example, nectar of the orchid *Epipactis helleborine* can contain as many as 100 compounds (Jakubska *et al*., 2005). In agriculture, models predicted that chemical combinations would be robust to resistance (Roush, 1998); empirically, broccoli plants with two *Bacillus thuringiensis* toxin genes were less prone than single‐toxin plants to the evolution of herbivore resistance (Zhao *et al*., 2003). Clinically, combination therapy is the recommended treatment for a number of diseases, including protozoan infections such as visceral leishmaniasis (*Leishmania donovani*) and malaria (*Plasmodium* spp.) (van Griensven *et al*., 2010), and has been proposed as an ‘optimal strategy’ to combat resistance (Bonhoeffer *et al*., 1997). In *Plasmodium falciparum*, resistance to the antimalarial drug artemisinin developed rapidly, but phytochemically complex *Artemisia annua* retained its medicinal value (Elfawal *et al*., 2015). Even if resistance does develop, it may have associated costs in the absence of inhibitory chemicals. These costs may limit the spatial spread and temporal persistence of resistance in populations when chemically mediated selective pressure is sporadic (Vanaerschot *et al*., 2014), as would be likely in diverse floral landscapes.

To assess whether a pollinator parasite can evolve resistance to single or combined floral phytochemicals under chronic exposure, we tested the ability of the bumble bee parasite, *C. bombi*, to evolve resistance to the naturally occurring antitrypanosomal floral phytochemicals thymol, eugenol and a thymol‐eugenol blend. We predicted that chronic exposure would (i) increase phytochemical resistance and (ii) decrease the growth‐inhibiting effects of a given phytochemical concentration, but that (iii) resistance would be slower or less likely to develop against the two‐phytochemical blend. In addition, we expected that (iv) resistance would come at a cost of decreased maximum growth in the absence of phytochemicals.

## Materials and methods

### Study system

*Crithidia bombi* is a trypanosome mid‐ and hindgut parasite of bumble bees (*Bombus* spp.) (Lipa & Triggiani, 1988; Sadd & Barribeau, 2013). *Crithidia bombi* is found on multiple continents (Schmid‐Hempel & Tognazzo, 2010), including in many species threatened by parasite‐related decline (Cameron *et al*., 2011; Schmid‐Hempel *et al*., 2014). *Crithidia bombi* lives in the intestinal tract of nectar‐ and pollen‐consuming bees, where it is directly exposed to the phytochemicals ingested by its hosts (Hurst *et al*., 2014). Although phytochemical concentrations in the gut lumen could be altered by microbial or host metabolism, orally transmitted parasites such as *C. bombi* are likely to have direct exposure to host‐ingested nectar and pollen phytochemicals in the crop, and possibly also in the mid‐ and hindgut. Parasites can also be exposed to phytochemicals at flowers themselves, which are sites of parasite transmission (Durrer & Schmid‐Hempel, 1994; Graystock *et al*., 2015). *Crithidia bombi* has several context‐dependent effects on host fitness (Sadd & Barribeau, 2013), and infection has been correlated with declining populations of native bees (Schmid‐Hempel *et al*., 2014). However, ingestion of phytochemicals may ameliorate infection (Manson *et al*., 2010; Baracchi *et al*., 2015; Richardson *et al*., 2015) and directly inhibit parasite growth (Palmer‐Young *et al*., accepted; E.C. Palmer‐Young, B.M. Sadd, R.E. Irwin, & L.S. Adler, In revision). The phytochemicals encountered by *C. bombi* are dependent on the spatially and temporally variable floral landscape utilized by bumble bees.

Thymol and eugenol are two widespread phytochemicals to which *C. bombi* can have prolonged exposure, either alone or in combination. Both of these phytochemicals have recognized antitrypanosomal effects (Santoro *et al*., 2007a, b), including against *C. bombi* (Palmer‐Young *et al*., accepted; E.C. Palmer‐Young, B.M. Sadd, R.E. Irwin, & L.S. Adler, In revision). Thymol occurs in a variety of floral honeys (Nozal *et al*., 2002; Viñas *et al*., 2006), but is most well documented in culinary herbs of the Lamiaceae, such as *Origanum vulgare* (oregano), *Origanum majorana* (marjoram), *Origanum dictamus* (dictamus) and *Thymus vulgaris* (common thyme) (Daferera *et al*., 2000), where thymol was recently quantified (5–8 ppm) in floral nectar (Palmer‐Young *et al*., accepted). Eugenol, or its derivative methyl eugenol, has been found in over 450 species from over 80 plant families (Tan & Nishida, 2012), including in the flowers of over 100 species (Tan & Nishida, 2012), making it one of the most common floral phytochemicals. Eugenol has been found in common crop species, such as *Cucurbita pepo* (Granero *et al*., 2005) and *Ocimum selloi* (Martins *et al*., 1997), ornamentals such as *Rosa rugosa* (Wu *et al*., 1985; Dobson *et al*., 1990), and wild *Epipactis* (Jakubska *et al*., 2005) and *Gymnadenia* (Gupta *et al*., 2014) orchids. Like thymol, eugenol is most extensively documented in plants of the Lamiaceae (38 species) (Tan & Nishida, 2012). In at least four Lamiaceae species, eugenol is found together with either thymol or thymol's isomer, carvacrol: *T. vulgaris* (Lee *et al*., 2005), *Ocimum basilicum* (Lee *et al*., 2005; Politeo *et al*., 2007), *O. vulgare* (Milos *et al*., 2000) and *O. majorana* (Deans & Svoboda, 1990).

The flowering periods of thymol‐ and eugenol‐rich plant species may expose pollinators and parasites to these phytochemicals for extended periods of time. The flowering period of thymol‐ and carvacrol‐rich *Thymus pulegioides* generally lasts for 1–2 months in late spring and early summer (Senatore, 1996), coinciding with maximal plant monoterpenoid content (Kaloustian *et al*., 2005); the flowering period of *T. vulgaris* may last for several months at lower latitudes (McGimpsey *et al*., 1994; Khazaie *et al*., 2008). Similarly, the flowering period of *O. basilicum* lasted 3 months in Poland, with *Bombus* spp. comprising 32% of visitors to the nectar‐ and pollen‐rich flowers (Chwil, 2007). In our experiments, we exposed parasites to phytochemicals for 6 weeks, to reflect both (i) the duration of flowering in thymol‐ and eugenol‐rich plants and (ii) the foraging lifetime of a *Bombus* worker, which typically specializes on a single floral species (Heinrich, 1976b).

### Parasite collection and culturing

*Crithidia bombi* cells were isolated from wild bumble bees (*Bombus impatiens*) collected near Normal, IL, United States, in 2013 (strain ‘IL13.2’, collected by BMS). The culture was established by flow cytometry‐based single cell sorting of bee faeces as described previously (Salathé *et al*., 2012). Cultures were microscopically screened to identify samples with strong *Crithidia* growth and absence of bacterial or fungal contaminants, then stored at −80 °C in a 2 : 1 ratio of cell culture: 50% glycerol until several weeks before the experiments began. Thereafter, cells were incubated in tissue culture flasks at 27 °C and propagated twice per week at a density of 100 cells μL^−1^ in 5‐mL fresh culture medium, the composition of which has been previously described (Salathé *et al*., 2012). The final transfer (to 500 cells μL^−1^ in 5 mL fresh medium) occurred 48 h before the experiment began.

### Phytochemicals

Thymol (Fisher Scientific, Franklin, MA, USA) and eugenol (Acros, Thermo Fisher, Franklin, MA, USA) stock solutions were prepared by pre‐dissolving phytochemicals in ethanol (thymol and eugenol: 10 * 10^3^ ppm for propagations, 40 * 10^3^ ppm for EC50 assays; blend: 10 * 10^3^ ppm thymol + 40 * 10^3^ ppm eugenol). Stock solutions were sterile‐filtered, aliquoted to sterile 2‐mL tubes and stored at −20 °C throughout each 6‐week experiment.

### Experimental design

We conducted three 6‐week exposure experiments, during which *C. bombi* was propagated continuously in either thymol (12 ppm), eugenol (50 ppm) or a 1 : 4 thymol:eugenol blend (5 ppm thymol + 20 ppm eugenol). Assuming a generation time of ~10 h (Salathé *et al*., 2012), the 6‐week exposure period corresponds to approximately 100 generations. Exposure concentrations were chosen to inhibit growth by approximately 50%. Because phytochemical composition of thymol‐ and eugenol‐containing plants varies across species, cultivars and seasons (Kaloustian *et al*., 2005; Lee *et al*., 2005; Wogiatzi *et al*., 2011), no single phytochemical ratio can encompass the variable proportions at which these compounds occur in plants. The 1 : 4 thymol:eugenol ratio was chosen to reflect the ratio of EC50 values for these two compounds in previous experiments (Palmer‐Young *et al*., accepted), such that each phytochemical would make approximately equal contribution to growth inhibition.

To initiate each of the three experiments, the ancestral *C. bombi* culture was divided into five phytochemical‐exposed and five control cell lines at an initial density of 100 cells μL^−1^ [adjusted using OD (optical density)] in 1 mL of the appropriate phytochemical‐containing medium (exposed lines) or phytochemical‐free medium (control lines). Sterile ethanol was added to control treatment medium to equalize ethanol concentrations in the two treatments (thymol experiment: 0.12% v/v, eugenol experiment: 0.5%, blend experiment: 0.05%). Cells were incubated at 27 °C in 12‐well plates inside zippered plastic sandwich bags to reduce the chance of contamination. Cells were transferred twice per week (100 cells μL^−1^ in 1‐mL treatment medium) for 6 weeks after 3 days (odd transfers: 3 days, 10 days, …, 39 days) or 4 days (even transfers: 7 days, 14 days, …, 42 days) of growth. An additional two transfers (45 days and 49 days) were made in the blend experiment, for a total exposure time of 49 days. Cell density at time of transfer – a measure of the amount of growth during the preceding incubation period – was estimated by measuring OD (630 nm) of a 200‐μL aliquot of each cell line. To obtain an accurate measure of cell density, the 12‐well plates containing the cells to be transferred were resuspended (30 s, 600 rpm) on a microplate shaker. The plates were then moved into a laminar flow cabinet, and 200 μL from each well of cultured cells, and also cell‐free control media containing the appropriate phytochemical concentration, was transferred to a 96‐well plate for spectrophotometric OD (630 nm) measurement. The difference in OD between the cultured cells and the cell‐free control media of corresponding phytochemical concentration was calculated for each sample. For analysis, OD readings were standardized relative to the mean OD of the control cell lines of the corresponding experiment and week.

The effects of the exposure treatment on phytochemical tolerance over time were assessed using three different response variables: (i) cell density at time of transfer, which tested the effects of a fixed phytochemical concentration to which the cells were chronically exposed, and (ii) EC50 (i.e. the phytochemical concentration that inhibited growth by 50%) from the weekly assays, which tested growth across a range of concentrations. In addition, to assess possible costs of resistance, we compared (iii) growth in phytochemical‐free control medium, a measure of the cost of resistance in exposed lines. These values reflect growth in wells with 0 ppm phytochemical during the EC50 assays (described below).

Note that for response variables (ii) and (iii) above, exposed and control lines were tested under the same respective conditions following 48‐h incubation in the absence of phytochemicals.

### EC50 assays

EC50 assays were conducted weekly on three of the five independently propagated cell lines from each treatment to determine the phytochemical concentration that inhibited growth by 50%. Each assay tested resistance to the same phytochemical or blend used in the exposure treatment, that is thymol EC50 for experiment testing effects of thymol exposure; eugenol EC50 for eugenol experiment; and 1 : 4 thymol : eugenol blend EC50 for the blend experiment. Six concentrations of the appropriate phytochemical (or blend), including a 0 ppm phytochemical control concentration, were prepared by two‐fold serial dilution in sterile‐filtered growth medium. The maximum concentrations used were 100 ppm w/v thymol (thymol experiment), 400 ppm eugenol (eugenol experiment) and 60 ppm thymol + 240 ppm eugenol (blend experiment). These concentrations resulted in nearly complete growth inhibition, which allowed accurate estimation of dose–response curves and EC50 values. Sterile ethanol was added to control treatment medium to equalize ethanol concentrations in all wells (thymol experiment: 0.25% v/v, eugenol experiment: 1%, blend experiment: 0.6%).

Two days before each week's EC50 assay began, an aliquot of cells from the lines propagated in 12‐well plates was transferred to fresh medium (5 mL) at a density of 500 cells μL^−1^. These cells were allowed to grow for 48 h in tissue culture flasks in the absence of phytochemicals. Immediately before the assay, each cell line was adjusted to a cell density of 1000 cells μL^−1^ in 8‐mL fresh medium. During weeks 0 and 1 of the thymol experiment, cell density was adjusted based on hand counting of *C. bombi* cells at 400× in a Neubauer hemocytometer. However, *C. bombi* swim quickly and were difficult to quantify. To more precisely equalize cell densities in subsequent assays, we adjusted cell density based on OD thereafter, using a predetermined linear correlation between cell counts and OD readings (cell density = 1.03 * 10^5^ * OD, *r* ^2^ = 0.93), where cell density is in cells μL^−1^ and OD is the difference in OD between the sample and an equivalent volume (200 μL) of control medium.

A separate 96‐well plate was prepared for each cell line. Each plate contained eight replicate wells at each of six phytochemical concentrations. To each well, 100 μL of 1000 cells μL^−1^ cell suspension was added to 100 μL of phytochemical‐enriched treatment medium using a multichannel pipette, resulting in a starting cell density of 500 cells μL^−1^. The outer wells of the plate were used for cell‐free controls (100 μL treatment medium +100 μL control medium) to control for changes in OD unrelated to cell growth. Plates were sealed with laboratory film and incubated inside zippered plastic sandwich bags for 5 days at 27 °C. Growth was measured by OD readings (630 nm) at 24‐h intervals. OD readings (630 nm) were taken immediately after resuspension of the cells on a microplate shaker (40 s, 1000 rpm, 3 mm orbit). We calculated net OD (i.e. the amount of OD resulting from parasite growth) by subtracting the average OD reading of cell‐free control wells of the corresponding concentration and time point.

For the thymol and blend analyses, we excluded the outermost two replicates (plate columns 3 and 10) of each concentration. Growth in these replicates differed from growth of the interior samples in the same treatments; we attributed this growth variation to volatility of the thymol, which resulted in altered exposure to phytochemicals depending on the contents of the neighbouring control wells. In the eugenol experiment, we excluded the final week's EC50 assays (i.e. time = 6 weeks) from analysis due to aberrantly hot laboratory conditions (40–43 °C, due to a building heating abnormality); cells were exposed to 40 °C temperatures for several hours during the set‐up of the assay and to 43 °C for an additional hour during the 24‐h growth reading.

### Statistical analyses

To quantify resistance to phytochemicals, EC50 values (i.e. the phytochemical concentrations that inhibited growth by 50% relative to phytochemical‐free controls) were interpolated by constructing separate dose–response curves of phytochemical concentration vs. growth for each cell line (*n* = 3 lines per treatment) and time point (*n* = 6–7 weeks per experiment). All statistical analysis was conducted using the open source software R v3.2.1 (R Core Team, 2014) following methods used for antimicrobial peptides (Rahnamaeian *et al*., 2015). Growth was quantified using the growth integral (i.e. area under the curve of net OD vs. time) for each well; this integral was calculated by fitting a model‐free spline to the observed OD measurements using grofit (Kahm *et al*., 2010). The relationship between phytochemical concentration and growth integral was modelled with a Markov chain Monte Carlo algorithm using Just Another Gibbs Sampler (Plummer, 2003) in combination with the R package rjags (Plummer, 2016). We used the following model to describe the relationship between phytochemical concentration (*c*) and growth integral (*g*):

(1) $$g=r\;-\;\frac{E_{\max}\;c^{h}}{\left({\left(C_{50}\right)}^{h}\;+\;c^{h}\right)}$$

where *r* denotes growth in the absence of the phytochemical, *E*_max_ represents the maximum inhibition at high concentrations, and *C*_50_ is the phytochemical concentration at which 50% of the maximum inhibition is reached. The parameter *h*, the Hill coefficient, indicates how steeply the inhibition increases around the concentration *C*_50_. From this model, we derived parameter estimates and 95% highest posterior density credible intervals (CI) of the EC50. For the blend experiment, in which all treatments contained a 1 : 4 thymol : eugenol ratio, curves were fitted using eugenol concentration as *c*. Growth measurements from the 0 ppm concentration were used to assess costs of resistance in the absence of phytochemicals.

To assess whether cell lines evolved resistance due to chronic phytochemical exposure, the effects of the exposure treatment over time were assessed using linear mixed‐effects regression models with the lmer function in R package lme4 (Bates *et al*., 2015). Each response variable (EC50, cell density at time of transfer and growth without phytochemicals) was standardized relative to the mean of the control lines at the corresponding time point. Exposure treatment and the treatment by time interaction were used as predictor variables, and cell line was included as a random effect to account for repeated measures. Significance of terms in the model was assessed by chi‐squared (χ^2^) tests with the anova function in the R package car (Fox & Weisberg, 2011). Fitted model means and standard errors were obtained using the lsmeans package (Lenth, 2016); graphs were produced with ggplot2 (Wickham, 2009) and cowplot (Wilke, 2016).

## Results

**Figure 1:**
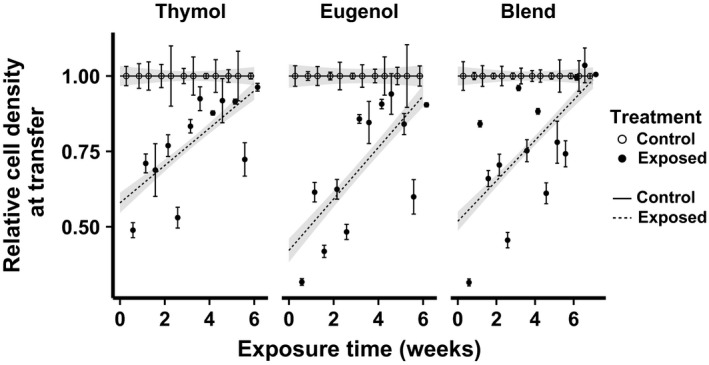
Chronic exposure of *Crithidia bombi* to phytochemicals decreased the growth‐inhibiting effects of the exposure treatments. The *x*‐axis shows the cumulative duration of exposure to phytochemical treatments. The *y*‐axis shows cell density at time of transfer [estimated using OD (630 nm)] after incubation in thymol (12 ppm), eugenol (50 ppm) or a thymol‐eugenol blend (5 ppm thymol + 20 ppm eugenol), standardized relative to the mean of the control lines at the corresponding time point. Points and error bars show mean ± SE (*n* = 5 lines per treatment). Lines and shaded bands show predicted means ± SE from linear mixed model fits. Open circles and solid lines: control treatment; filled circles and dashed lines: phytochemical exposure treatment.

Chronic phytochemical exposure resulted in increased phytochemical resistance in all three experiments. Changes in cell density at time of transfer indicated remarkably increased resistance to phytochemicals. In each experiment, the highly significant Treatment: Week interaction (Table 1) indicated that the growth‐inhibiting effect of the fixed‐concentration exposure treatment decreased over the course of the exposure period. Initially, the phytochemical exposure treatments (12 ppm thymol, 50 ppm eugenol or 5 ppm thymol + 20 ppm eugenol) inhibited growth by over 50% (Fig. 1). However, by the end of the 6‐week experiment, the same phytochemical concentration had minimal effect on parasite growth in the lines that were chronically exposed to the phytochemical treatment. In other words, after 6 weeks, the exposed lines grew nearly as fast in the presence of phytochemicals as the controls grew in the absence of phytochemicals.

**Table 1:** Effects of exposure treatments on *Crithidia bombi* cell density at time of transfer [estimated using OD (optical density) at 630 nm], EC50 and growth in the absence of phytochemicals. All responses were standardized relative the mean of the control lines of the corresponding experiment and time point. Predictor variables of linear mixed models were tested for statistical significance using χ^2^ tests

Exposure treatment	Predictor	χ^2^	d.f.	*P*
Relative cell density at time of transfer				
Thymol	Treatment	**80.29**	**1**	**< 0.001**
	Treatment: Week	**46.41**	**2**	**< 0.001**
Eugenol	Treatment	**111.27**	**1**	**< 0.001**
	Treatment: Week	**80.40**	**2**	**< 0.001**
Blend	Treatment	**116.48**	**1**	**< 0.001**
	Treatment: Week	**80.65**	**2**	**< 0.001**
Relative EC50				
Thymol	Treatment	2.16	1	0.14
	Treatment: Week	**19.16**	**2**	**< 0.001**
Eugenol	Treatment	2.09	1	0.15
	Treatment: Week	**9.96**	**2**	**0.01**
Blend	Treatment	2.95	1	0.09
	Treatment: Week	**7.45**	**2**	**0.02**
Relative growth without phytochemicals				
Thymol	Treatment	**14.95**	**1**	**< 0.001**
	Treatment: Week	**39.48**	**2**	**< 0.001**
Eugenol	Treatment	1.5874	1	0.21
	Treatment: Week	**35.2**	**2**	**< 0.001**
Blend	Treatment	2.18	1	0.14
	Treatment: Week	5.53	2	0.06

Bold: *P *<* *0.05.

**Figure 2:**
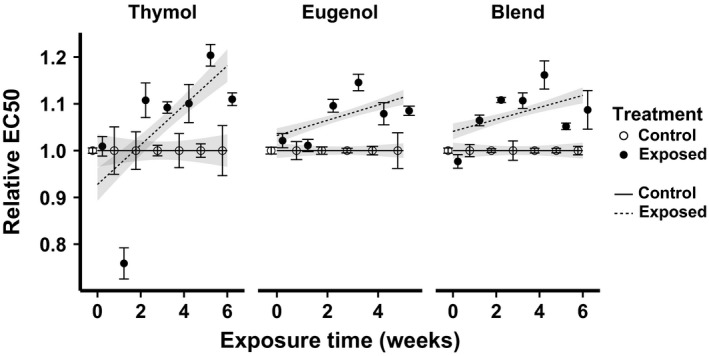
Phytochemical exposure treatments increased phytochemical resistance in *Crithidia bombi*. The *x*‐axis shows the cumulative duration of exposure to phytochemical treatments. The *y*‐axis shows EC50 (phytochemical concentration resulting in 50% of maximal growth inhibition), standardized relative to the mean of the control lines at the corresponding time point. Points and error bars show mean ± SE (*n* = 3 lines per treatment). Lines and shaded bands show predicted means ± SE from linear mixed model fits. Open circles and solid lines: control treatment; filled circles and dashed lines: phytochemical exposure treatment.

Because the changes in cell density could have reflected both environmental acclimation and genetic changes, we also conducted weekly EC50 assays following a brief relaxation of selection (48‐h growth in phytochemical‐free media) to minimize contributions of the parental environment to the resistance phenotype. From the 2‐week assay onward, EC50 values in the exposed lines were consistently higher than those of controls (Fig. 2). Thymol exposure increased resistance to thymol; eugenol exposure increased resistance to eugenol; and exposure to a 1 : 4 thymol‐eugenol blend increased resistance to the same 1 : 4 blend. For each experiment, the Treatment: Week interaction term was highly significant (Table 1); this indicates that the EC50 ratio between exposed and control lines increased over the exposure period. Increases in EC50 relative to the control were similar across the three experiments (~10%, Fig. 2).

**Figure 3:**
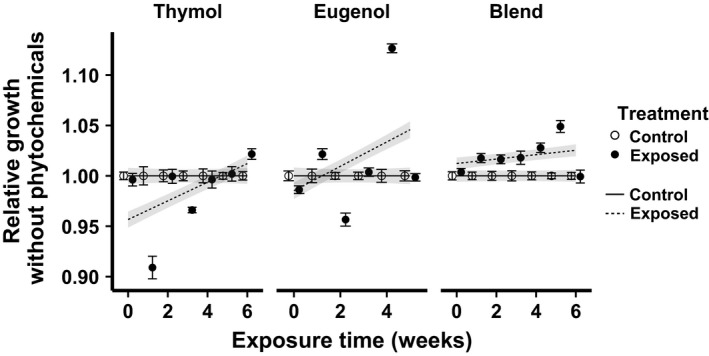
Growth without phytochemicals (i.e. at phytochemical concentration of 0 ppm) during each week's EC50 assays. The *x*‐axis shows the cumulative duration of exposure to phytochemical treatments. The *y*‐axis depicts growth in the absence of phytochemicals, standardized relative to the mean of the control lines at the corresponding time point. Points and error bars show mean ± SE [*n* = 6 (thymol and blend) or eight (eugenol) wells each of three lines per treatment]. Lines and shaded bands show predicted means ± SE from linear mixed model fits. Open circles and solid lines: control treatment; filled circles and dashed lines: phytochemical exposure treatment.

We found little evidence for costs of adaptation in terms of reduced growth in the absence of inhibitory phytochemicals. In the thymol experiment, there was an initial negative effect of the exposure treatment on growth without phytochemicals, but also a significant amelioration of this negative effect over time (Treatment: Week interaction, Table 1; Fig. 3). However, this result was strongly driven by the poor growth in exposed lines at the 1‐week time point. When the 1‐week time point was removed from the model, the negative effect of treatment was no longer significant (χ^2^ = 2.23, d.f. = 1, *P *=* *0.14). However, there remained a significant positive Treatment: Week interaction (χ^2^ = 8.58, d.f.  = 2, *P* = 0.013), indicating progressively better growth relative to controls over time. Across all weeks, growth of thymol‐exposed lines in the absence of phytochemicals averaged 98.6% that of controls, or 99.7% after excluding the 1‐week time point. In the eugenol experiment, there was again no significant effect of treatment; across all weeks, growth in the absence of phytochemicals differed by only 0.4% between treatments. As in the thymol experiment, there was again a positive Treatment: Week interaction, which was statistically significant, but inconsistent across time (Fig. 3). In the blend experiment, exposed lines tended to have nonsignificantly higher growth without phytochemicals than the controls (*P* = 0.14, Table 1), and there was a marginally significant tendency of increased relative growth over time (*P* = 0.06, Table 1).

## Discussion

We tested the effects of chronic phytochemical exposure on the evolution of resistance by the bumble bee parasite *C. bombi* in cell culture. The parasite evolved comparable resistance to both single phytochemicals and a two‐compound combination, and resistance had no growth‐related costs under phytochemical‐free conditions. Thus, chronic exposure to ecologically relevant levels of floral phytochemicals could lead to the evolution of parasite resistance that may weaken the medicinal effects of phytochemicals on pollinators.

### Chronic phytochemical exposure increased resistance

Initially, phytochemical exposure treatments inhibited *C. bombi* growth by > 50%; however, after 6 weeks of exposure, the same phytochemical concentrations resulted in minimal inhibition (Fig. 1). Our thymol exposure concentration (12 ppm) exceeded levels in *T. vulgaris* nectar [5.2–8.2 ppm thymol (Palmer‐Young *et al*., accepted)] and honey from thymol‐fumigated honey hives [7.5 ppm (Charpentier *et al*., 2014)]. Similarly, our eugenol exposure concentration (50 ppm) equalled concentrations in *Rosa x hybrida* stamens (Bergougnoux *et al*., 2007), but far exceeded concentrations in other flowers and honey (Palmer‐Young *et al*., accepted). In other words, within a few weeks, parasites became almost completely resistant to the effects of naturally occurring levels of phytochemicals.

Exposure over the 6‐week time frame used in our experiments is plausible in natural systems. For example, *O. basilicum*, which can contain both thymol and eugenol (Lee *et al*., 2005; Politeo *et al*., 2007), flowers for a 3‐month period, even in northern Europe (Chwil, 2007), and its nectar and pollen are highly attractive to bumble bees. Individual *Bombus* workers, which live for four to 6 weeks, tend to specialize on particular plant species (Heinrich, 1976b). Thus, in a worker that specializes on a plant rich in one or several phytochemicals, resident parasites would have ample time to evolve resistance within a single growing season.

We expect that our serial propagation experiments provide a conservative estimate of the ability of natural parasite populations to evolve phytochemical resistance. In contrast to the low initial diversity of our clonal parasite cell lines, *C. bombi* populations are genetically diverse (Tognazzo *et al*., 2012), and phytochemical resistance can vary severalfold between genotypes (Palmer‐Young *et al*., accepted). High levels of pre‐existing natural variation could result in even more dramatic responses to selection than what we observed using clonal cell lines. Conversely, however, exposure of parasites in nature to nutrient limitation or host immune responses could increase parasite generation times, thereby slowing evolutionary processes and reducing rates of phytochemical adaptation.

### Combined phytochemicals did not curtail the evolution of resistance

Contrary to our prediction, a two‐phytochemical combination of thymol and eugenol did not inhibit the evolution of resistance. This is incongruent with empirical studies (Zhao *et al*., 2003; Elfawal *et al*., 2015), theoretical predictions (Roush, 1998) and clinical recommendations (van Griensven *et al*., 2010), all of which suggest that resistance should evolve more slowly to blends than to single compounds. Our result may relate to interactions between thymol and eugenol and to their modes of action. First, we have found synergistic effects of thymol and eugenol against *C. bombi* growth (E.C. Palmer‐Young, B.M. Sadd, R.E. Irwin, & L.S. Adler, In revision), which may have promoted evolution of resistance by increasing the marginal benefits of resistance to either compound (Yeh *et al*., 2009). Second, the similar pro‐oxidant modes of action of thymol and eugenol may have facilitated simultaneous development of resistance against both compounds. Both the monoterpenoid thymol and the phenylpropanoid eugenol are lipophilic compounds with aromatic rings and free hydroxyl groups. Such compounds penetrate membranes, disrupt ionic gradients and energy production and increase oxidative stress (Bakkali *et al*., 2008). Trypanosomes can counteract oxidative stress by producing thiols (Mehlotra, 1996), heat shock proteins (McCall & Matlashewski, 2012) and glycerol (Husain *et al*., 2012). In *L. donovani*, these antioxidant systems can be quickly up‐regulated by increasing expression of antioxidant enzymes and even duplication of antioxidant‐encoding chromosomes (Mannaert *et al*., 2012), resulting in rapid development of resistance against pro‐oxidant drugs (Vanaerschot *et al*., 2014). *Crithidia bombi* encounters pro‐oxidant floral phytochemicals, osmotic stress and UV radiation during transmission at flowers (Cisarovsky & Schmid‐Hempel, 2014), and had extremely high resistance to phenolics relative to clinically important trypanosomes (Palmer‐Young *et al*., accepted). Therefore, *C. bombi* likely possesses extensive antioxidant mechanisms that could facilitate rapid adaptation to pro‐oxidant phytochemicals. If particular genotypes have broad‐spectrum resistance against multiple phytochemicals with similar modes of action, resistance to one phytochemical could confer resistance to other phytochemicals as well.

### No apparent growth‐related cost of resistance in the absence of phytochemicals

The spread and maintenance of chemical resistance in parasite populations is shaped by a balance between the strength of selection favouring resistance and the costs of resistance that favour competing susceptible genotypes (Lenormand, 2002). In our experiments, we found no evidence for resistance‐related costs in terms of growth under phytochemical‐free conditions. Our previous work, which showed extremely high phytochemical resistance of *C. bombi* relative to related trypanosomes (Palmer‐Young *et al*., accepted), suggests that phytochemical‐resistant strains of *C. bombi* have indeed been quite successful in nature. Although drug resistance appears to be costly in *Plasmodium* spp. and schistosomes (Vanaerschot *et al*., 2014), no costs of paromycin resistance were found in *L. donovani* (Hendrickx *et al*., 2015); in *L. infantum*, miltefosine resistance was costly, but paromycin resistance resulted in increased growth and enhanced tolerance to stress (Hendrickx *et al*., 2016). Resistance to pro‐oxidant antimonial drugs can actually improve *L. donovani* infectivity and establishment in hosts, presumably because the superior antioxidant defences of resistant lines allow them to tolerate host immune responses and the stress of initial establishment (Vanaerschot *et al*., 2011). The fitness advantages of chemical resistance in parasites may also be context‐dependent. For example, drug‐resistant and drug‐susceptible *L. donovani* competed equally well under optimal conditions, but drug‐resistant lines outcompeted susceptible lines under stressful conditions, including heat shock, pH change, starvation and infection of host cells (García‐Hernández *et al*., 2015). If phytochemical‐resistant *C. bombi*, like resistant *L. donovani*, gain a competitive advantage under temperature‐ or food‐stressed conditions, then chemically resistant parasites could be favoured in communities of stressed or resource‐limited pollinators. Food‐stressed bees are already immunocompromised (Brunner *et al*., 2014) and more vulnerable to *C. bombi*‐induced mortality (Brown *et al*., 2003). Moreover, immunocompromised hosts could promote the spread of chemically resistant parasites by failing to eradicate residual parasites following chemical treatment (Bloland, 2001), thereby allowing chemically resistant parasites to survive and spread to new hosts. As a result, the spread of phytochemical‐resistant *C. bombi* may be most favoured under conditions when host bees are most susceptible to infection.

### Ecological determinants of resistance to phytochemicals

Although *C. bombi* can evolve resistance to phytochemicals and blends without incurring apparent costs, several factors may constrain parasite adaptation to local phytochemicals in wild populations, thus maintaining submaximal phytochemical resistance that varies among strains (Palmer‐Young *et al*., accepted). These factors could include complex and varied phytochemical environments, high rates of migration, periodic population bottlenecks and possible transmission‐related costs of resistance. First, nectar and pollen contain a rich diversity of phytochemicals. For example, more than 60 compounds, including thymol and eugenol, were present in floral essential oils of *Helichrysum arenarium* (Lemberkovics *et al*., 2001), and over 100 compounds, including eugenol, were found in nectar of the orchid *E. helleborine* (Jakubska *et al*., 2005). As shown in experiments with *A. annua* and *P. falciparum* malaria (Elfawal *et al*., 2015), it may be difficult for parasites to adapt to these complex blends, particularly when bees consume a mixture of blends from different types of flowers. Second, migration of parasites between different types of landscapes could limit local adaptation. Bumble bees forage over many kilometres (Heinrich, 2004), and founding queens may disperse considerable distances to found new colonies, thereby homogenizing parasite populations from regions with different floral phytochemical characteristics. Furthermore, sexual reproduction in *C. bombi* could increase the frequency of recombination events (Schmid‐Hempel *et al*., 2011) that break up resistance‐conferring gene complexes. Third, genetic drift may limit the influence of natural selection on *C. bombi* populations by imposing annual genetic bottlenecks. Unlike honey bee colonies, bumble bee colonies in temperate climates have an annual cycle and are founded anew each year by queens that mate in autumn, hibernate through the winter, and emerge in spring. Because queens alone survive the winter, and only a small proportion of queens succeed in founding colonies, *C. bombi* populations can be severely reduced between fall and spring (Erler *et al*., 2012), with possible random loss of resistance alleles. There may also be subtle costs of resistance that were undetectable in cell cultures. For example, costs related to between‐host transmission or within‐host growth could reduce the fitness of phytochemically resistant strains in the wild. Any combination of these factors could explain the maintenance of susceptibility to thymol and eugenol in *C. bombi* populations.

Despite the possibility that migration and genetic drift could weaken the effects of natural selection for phytochemical resistance, *C. bombi* does appear to have evolved extensive resistance to the nectar phenolic compounds caffeic, chlorogenic and gallic acids (Palmer‐Young *et al*., accepted). We hypothesize that parasites may be more likely to have chronic exposure to these compounds, which are prevalent at considerable concentrations in honey from the nectar of many floral species (Can *et al*., 2015). Although eugenol in particular is widespread in flowers, both thymol and eugenol are more volatile than the aforementioned phenolics, which may limit the duration of parasite exposure to these compounds. However, repeated prophylactic fumigation of hives with thymol – a common pest‐control measure for honey bee hives (Gregorc & Planinc, 2005) – could result in intense and prolonged selection for resistant parasites.

The distribution of phytochemicals in modern landscapes may contribute to evolution of phytochemical resistance. Sequential exposure to single chemicals is known to promote resistance (Bonhoeffer *et al*., 1997). Bees in agricultural settings may have sequential access to diets dominated by a single plant species during each period of the growing season (Goulson *et al*., 2015), which could give parasites ample time to adapt to each plant's phytochemicals. If phytochemical resistance in *C. bombi* is minimally costly and stable in the absence of phytochemicals – as observed in *L. donovani* (dos Santos *et al*., 2008; Hendrickx *et al*., 2012) – resistance could be maintained between annual periods of exposure to phytochemicals of particular floral species. Progressive augmentation of resistance to each agricultural species’ phytochemicals would decrease the medicinal value of phytochemicals for pollinators.

In contrast to monotony, diversity among plants and hosts could curtail the evolution of phytochemical resistance. Serial infection of related hosts could select for parasites with specialized resistance to the phytochemicals in the host's preferred food plants. However, transmission of parasites among bumble bee host species with different diets (Goulson & Darvill, 2004) could result in continually varying selective pressures that interrupt the development of phytochemical resistance. Because different pollinators favour different floral species (Heinrich, 1976a; Goulson & Darvill, 2004), pollinator and plant diversity could be mutually stabilizing. Diverse flora may also disrupt the development of resistance by exposing parasites to hundreds of phytochemicals simultaneously (Jakubska *et al*., 2005), rather than the two phytochemicals used in our thymol/eugenol blend. In addition to possible mitigation of phytochemical resistance among parasites, phytochemically and taxonomically varied landscapes have other known benefits to pollinators. Although thymol and eugenol are relatively benign and even attractive to bees (Goyret & Farina, 2005; Ebert *et al*., 2007), consumption of other potentially antiparasitic phytochemicals can increase mortality in bumble bees and other insects (Thorburn *et al*., 2015; Tao *et al*., 2016). Given that bumble bees are generalist pollinators, we hypothesize that they may be less susceptible to toxicity when allowed to consume mixed diets that do not contain excessive amounts of any particular compound. Furthermore, varied landscapes are more likely to provide the variety of nutrients needed for colony growth and development, and also to offer a temporally distributed supply of nectar and pollen throughout the growing season (Roulston & Goodell, 2011). Overall, whereas limited floral diversity may decrease pollinator diversity and streamline the evolution of phytochemical resistance, abundant floral diversity could reduce parasite resistance to any particular suite of phytochemicals.

## Conclusion

Our experiments show that pollinator parasites can evolve resistance to growth‐inhibiting floral phytochemicals without associated costs of reduced growth. In contrast to our predictions, resistance was not hindered by a two‐phytochemical combination. Given the initially low diversity of our parasite cell lines, these findings represent a conservative estimate of the ability of wild parasite populations to adapt to phytochemicals, a process that could diminish the value of naturally occurring defences against parasites. Low floral and host diversity can be expected to promote phytochemical resistance. If resistance is not costly, or even confers a fitness advantage, resistance traits could spread quickly, exacerbating vulnerability to infection in already threatened pollinators.

Data deposited in the Zenodo repository: URL: https://zenodo.org/record/54705 with restricted access for reviewers (Palmer‐Young *et al*., 2016); at acceptance, data will be made freely available.
